# *Phaeoisarialaianensis* (Pleurotheciales, Pleurotheciaceae), a new species from freshwater habitats in China

**DOI:** 10.3897/BDJ.10.e94088

**Published:** 2022-10-27

**Authors:** Yu Liu, Gui-Ping Xu, Xin-Yi Yan, Min-Hui Chen, Yang Gao, Hai-Jing Hu, Hai-Yan Song, Dian-Ming Hu, Zhi-Jun Zhai

**Affiliations:** 1 College of Bioscience and Bioengineering, Jiangxi Agricultural University, Nanchang, China College of Bioscience and Bioengineering, Jiangxi Agricultural University Nanchang China; 2 Bioengineering and Technological Research Centre for Edible and Medicinal Fungi, Jiangxi Agricultural University, Nanchang, China Bioengineering and Technological Research Centre for Edible and Medicinal Fungi, Jiangxi Agricultural University Nanchang China; 3 Key Laboratory of Crop Physiology, Ecology and Genetic Breeding (Jiangxi Agricultural University), Ministry of Education of the P. R. China, Nanchang, China Key Laboratory of Crop Physiology, Ecology and Genetic Breeding (Jiangxi Agricultural University), Ministry of Education of the P. R. China Nanchang China

**Keywords:** Ascomycota, *Phaeoisaria*, morphology, phylogenetic anaysis, taxonomy

## Abstract

**Background:**

Freshwater fungi play an indispensable role in the ecosystem and have great research value. Based on morphological and phylogenetic analyses of a concatenated dataset of ITS, LSU and SSU sequences, a new species, *Phaeoisarialaianensis*, was introduced as a freshwater hyphomycete from Anhui Province, China.

**New information:**

*Phaeoisarialaianensis* was morphologically described as erect, rigid, dark brown to black, velvety synnemata which has macronematous, septate, branched, brown to dark brown, parallel adpressed conidiophores with polyblastic, integrated, terminal, hyaline to pale brown, smooth, denticulate, sympodial conidiogenous cells and ellipsoidal to obovoid, rounded at the apex, obtuse and tapering towards base, septate, guttulate conidia. Based on molecular and morphological characteristics, it is confirmed to be a new species. All illustrations and descriptions have been provided.

## Introduction

*Phaeoisaria* (Pleurotheciales) was established by Höhnel (1909) to accommodate *Phaeoisariabambusae* as the type species, a hyphomycetous taxon isolated from a bamboo substrate. This genus is characterised by indeterminate synnemata with parallel adpressed conidiophores with numerous sympodially extending denticulate conidiogenous cells and aseptate or septate ellipsoidal, obovoidal, fusiform-cylindrical to falcate, hyaline conidia (Höhnel 1909, Réblová et al. 2016, Hyde et al. 2018, Luo et al. 2018, Boonmee et al. 2021). Nevertheless, indeterminate synnemata have not been observed in some species, such as *P.curvata* (de Hoog and Papendorf 1976), *P.glauca* (de Hoog and Papendorf 1976), *P.loranthacearum* (Crous et al. 2015), *P.fasciculata* (Réblová et al. 2016), *P.annesophieae* (Crous et al. 2017) and *P.dalbergiae* (Crous et al. 2021).

In the past decades, an increasing number of new species was assigned to *Phaeoisaria* by distinguishing characters (Crous et al. 2017, Hyde et al. 2018, Hyde et al. 2019, Luo et al. 2019, Boonmee et al. 2021, Crous et al. 2021). Until now, 26 species have been accepted in the genus *Phaeoisaria* (http://www.speciesfungorum.org/Names/Names.asp). These species are relatively common and have a worldwide distribution, while only four of them have been recorded in China. Moreover, there are presently only 15 species having the molecular data in *Phaeoisaria*. In this study, we depicted a new species, *Phaeoisarialaianensis*, from submerged wood in Anhui Province of China, with both morphological examination and molecular phylogenetic analysis.

## Materials and methods

### Samples collection, specimen examination and isolation

Submerged rotting wood samples were gathered from Laian County, Anhui Province, China and were brought back to the laboratory to be incubated in plastic boxes at room temperature. Fungi on the host surface were observed with a Nikon SMZ-1270 microscope (Nikon Corporation, Japan) and morphologically photographed with a Nikon ECLIPSE Ni-U compound microscope (Nikon Corporation, Japan), which was equipped with a Nikon DS-Fi3 camera. The structure of fungi was determined by PhotoRuler 1.1.3.0 (The Genus Inocybe, Hyogo, Japan) and figures were processed by Adobe Photoshop 2020 (Adobe Systems, USA). According to the method of Li et al. (2021), single spore isolation and pure culture were carried out. Fungal specimens were deposited in the Fungus Herbarium, Jiangxi Agricultural University, Nanchang, China.

### DNA extraction, PCR amplification and sequencing

By using the improved CTAB method (Doyle and Doyle 1987), fungal total genomic DNA was extracted from fresh mycelium. Three gene regions (ITS, LSU and SSU), were respectively amplified by polymerase chain reaction (PCR) using the primers of ITS1/ITS4 (White et al. 1990), LROR/LR7 (Hopple and Vilgalys 1999) and NS1/NS4 (White et al. 1990), with 25 μl of the final volume including 9.5 μl ddH_2_O, 12.5 μl 2× Taq PCR MasterMix (Qingke, Changsha, China), 1 μl of DNA template and 1 μl of each primer (10 μM). Then amplifications were conducted under the PCR conditions described by Zhai et al. 2022. The PCR products were purified and the sequencing reactions were commercially conducted with the corresponding forward and reverse primers by QingKe Biotechnology Co. (Changsha, China). All sequences were edited with SeqMan v. 7.1.0 (DNASTAR, lnc, Madison, WI) and were deposited in the NCBI GenBank database.

### Phylogenetic analysis

The sequences of 69 strains were retrieved from recent articles (Luo et al. 2018, Hyde et al. 2019, Boonmee et al. 2021) and downloaded from GenBank (Table 1). Each matrix of ITS, LSU and SSU was aligned using the online service of MAFFT v.7 (http://mafft.cbrc.jp/alignment/server/large.html, Katoh et al. 2019) and then the sequences of three regions were concatenated by PhyloSuite v.1.2.2 (Zhang et al. 2020). By using RAxML v.7.2.6 (Stamatakis and Alachiotis 2010), Maximum Likelihood (ML) analysis was performed, which used a GTRGAMMA substitution model with 1000 bootstrap replicates. The Markov Chain Monte Carlo (MCMC) method in MrBayes was used to estimate the posterior probabilities (PP) (Zhaxybayeva and Gogarten 2002) and it was set as four chains (2 hot chains and 2 cold chains) running 2,000,000 generations synchronously, resulting in 40002 trees in total. Based on the initial 25% of sampled data being cut off as burn-in, PhyloSuite v.1.2.2 (Zhang et al. 2020) was used to infer Bayesian inference phylogeny under the JC+I+G+F model of the concatenation of ITS, LSU and SSU. After visualisation by FigTree v.1.4.4 (Rambaut 2018), the phylogenetic tree was edited and illustrated using Adobe Illustrator 2020 (Adobe Systems Inc., USA). The aligned matrices and trees were submitted to TreeBASE (http://purl.org/phylo/treebase/phylows/study/TB2:S29791).

## Taxon treatments

### Phaeoisaria laianensis

Y. Liu, G.P. Xu, X.Y. Yan, D.M. Hu & Z.J. Zhai sp. nov.

2EE81D2F-85F9-51D4-9628-863AEF4186AB

844773

#### Materials

- **Type status:** Holotype. **Occurrence:** recordedBy: Yu Liu; occurrenceID: 99B9C819-CA87-5634-AB08-7E7A79E1ADE0; **Taxon:** scientificName: *Phaeoisarialaianensis*; acceptedNameUsage: *Phaeoisarialaianensis* Y. Liu, D.M. Hu & Z.J. Zhai; kingdom: Fungi; phylum: Ascomycota; class: Sordariomycetes; order: Pleurotheciales; family: Pleurotheciaceae; genus: Phaeoisaria; specificEpithet: *laianensis*; taxonRank: species; verbatimTaxonRank: species; scientificNameAuthorship: Y. Liu, D.M. Hu & Z.J. Zhai; **Location:** continent: Asia; country: China; stateProvince: Anhui; county: Laian; locality: Wawuzhuang; verbatimElevation: 35; locationRemarks: Label transliteration; verbatimCoordinates: 32.66 N, 118.65 E; verbatimLatitude: 32.66; verbatimLongitude: 118.65; **Identification:** identifiedBy: Yu Liu and Zhi-jun Zhai; dateIdentified: 2021; **Event:** samplingProtocol: collecting; eventDate: 06-05-2021; year: 2021; month: 5; day: 6; habitat: Freshwater; **Record Level:** type: PhysicalObject; language: en; rightsHolder: Dian-Ming Hu and Zhi-jun Zhai; institutionID: HFJAU10040; collectionID: LKJ17; institutionCode: the Herbarium of Fungi, Jiangxi Agricultural University (HFJAU); collectionCode: Fungi; ownerInstitutionCode: HFJAU

#### Description

**Figure 1.**
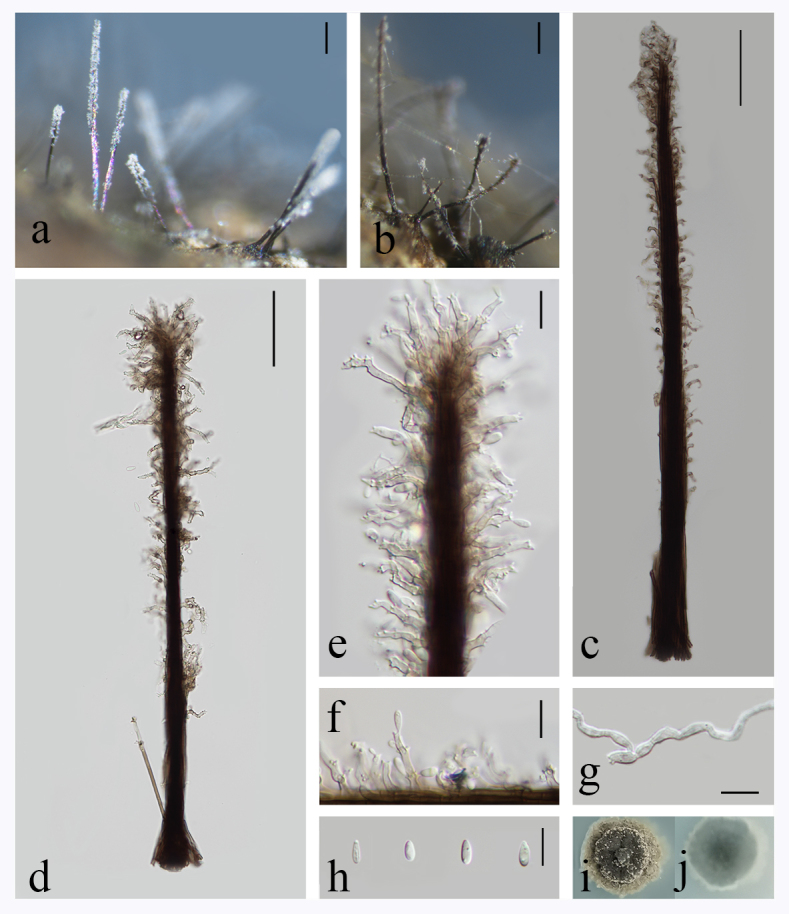
*Phaeoisarialaianensis* (HFJAU 10040, Holotype) **a, b** Colonies on wood; **c, d** Conidiophores; **e, f** Conidiogenous cells with conidia; **g** Germinating conidium; **h** Conidia; **i, j** Colony on PDA for 26 days from above and reverse. Scale bars: a, b = 100 µm, c, d = 50 µm, e–h = 10 µm.

Saprobic on decaying wood submerged in freshwater habitats. **Sexual morph**: Undetermined. **Asexual morph**: *Colonies* effuse, solitary, scattered, dark brown to black, hairy, covered by white conidial mass. *Mycelium* partly superficial, partly immersed. *Synnemata* 290–848 × 9.3–30.7 µm (x̅ = 532 × 18.6, SD = 159 × 5, n = 20), erect, rigid, dark brown to black, velvety, smooth, composed of compactly and parallel adpressed conidiophores. *Conidiophores* 116.2–491.1 × 2–3.2 µm (x̅ = 276.1 × 2.4, SD = 96.7 × 0.5, n = 10), macronematous, synnematous, septate, branched, brown to dark brown, smooth. *Conidiogenous cells* 8.3–27.5 × 2.3–3.8 µm (x̅ = 17.1 × 2.7, n = 10), polyblastic, integrated, terminal, hyaline to pale brown, smooth, denticulate, sympodial, each with several denticulate conidiogenous loci, 0.8–1.6 × 0.4–0.8 µm (x̅ = 1.3 × 0.7, n = 10). *Conidia* 5–7.2 × 1.7–2.9 µm (x̅ = 5.9 × 1.7, SD = 0.5 × 0.3, n = 50), ellipsoidal to obovoid, straight, rounded at the apex, obtuse and tapering towards base, hyaline, aseptate, guttulate, smooth-walled. (Fig. 1).

**Culture characteristics**: Conidia germinated within 24 h in which germ tubes were produced from both ends or sides at 28℃ on PDA. The colony on PDA grows up slowly and reaches 24.5 mm in 26 days, periphery grey, surface folded, middle grey-green to black, raised with mycelium in the centre, covered with lots of white conidia, powdery, reverse grey to black.

**Material examined**: China, Anhui Province, alt. 35 m, near 32.66°N, 118.65°E, on decaying wood submerged in a freshwater stream, 6 May 2021, Y. Liu, G.P. Xu and Z.J. Zhai, LKJ17 (HFJAU 10040, holotype), ex-type living culture, CCTCC AF 2022069 = CCTCC AF 2022073.

#### Etymology

The name reflects the district where this fungus was found.

#### Notes

**Figure 2.**
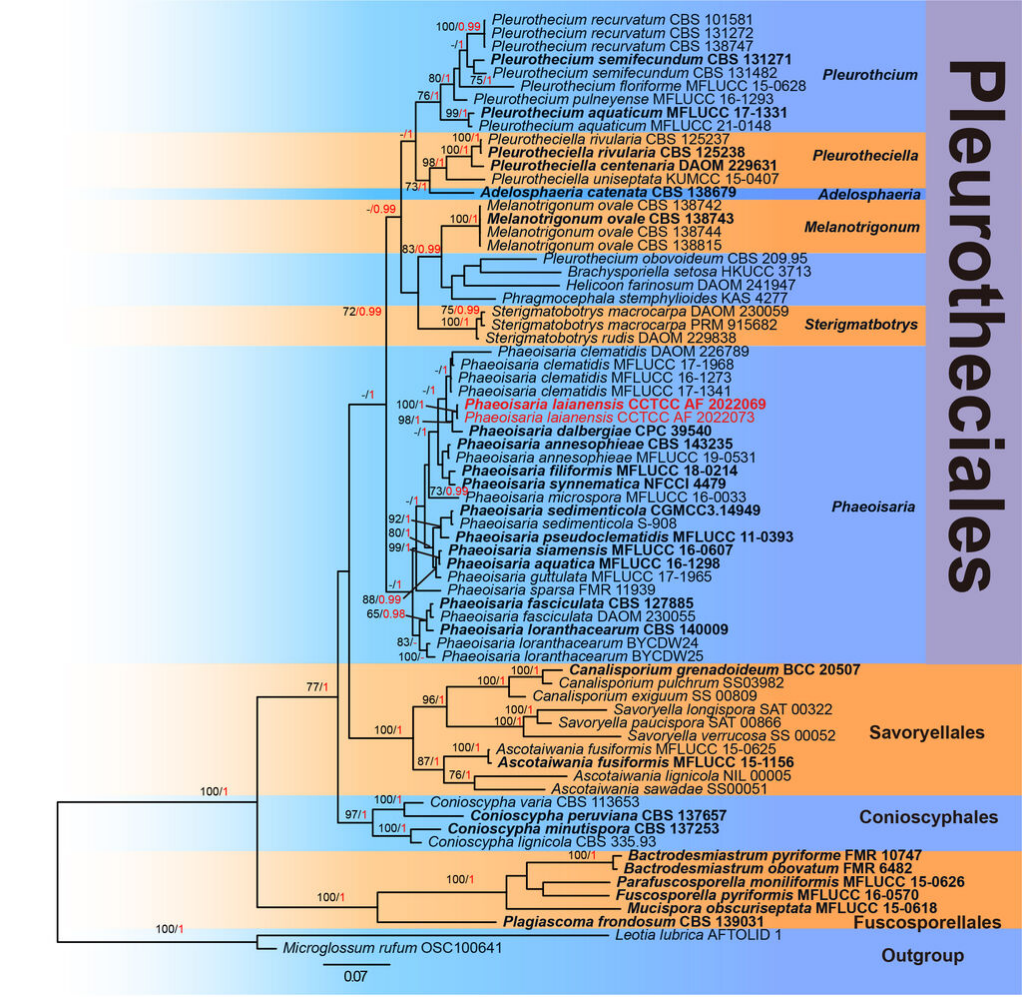
Phylogenetic tree of Bayesian analysis, based on a concatenated alignment of ITS, LSU and SSU sequences. Branch support is shown at the nodes, Maximum Likelihood bootstrap support (BS, black) ≥ 60% and Bayesian posterior probability (PP, red) ≥ 0.95. *Leotialubrica* (AFTOLID 1) and *Microglossumrufum* (OSC100641) are selected as the outgroup taxa. The new species is marked in red and ex-type strains are in bold.

Phylogenetic analysis shows that *Phaeoisarialaianensis* is a phylogenetically-distinct species, most closely related to *P.dalbergiae* and then to *P.clematidis* (Fig. 2). However, *P.laianensis* is easily distinguished from *P.dalbergiae* by its ellipsoidal to obovoid, rounded at the apex and tapering towards base conidia (Crous et al. 2021). In addition, *P.laianensis* has synnemata, which is absent in *P.dalbergiae* (Crous et al. 2021), also in *P.curvata*, *P.glauca* (de Hoog and Papendorf 1976), *P.loranthacearum* (Crous et al. 2015), *P.annesophieae* and *P.fasciculata* (Réblová et al. 2016) (Table 2). The new species is similar to *P.clematidis* in having resembling synnemata or conidia (Hughes 1958, Luo et al. 2018), while the former has shorter synnemata (290–848 µm vs. 1000–1500 µm) and smaller conidia (5–7.2 µm wide vs. 4–10 µm wide) than *P.clematidis* (Table 2). Likewise, *P.laianensis* has longer synnemata than *P.siameneis* (290–848 µm vs. 330–380 µm), smaller conidiophores than *P.guttulata* (Hyde et al. 2018) and *P.aquatica* (116.2–491.1 × 2–3 µm vs. 480–700 × 2–5 µm and 1028–1262 × 3.5–4.5 µm) (Luo et al. 2018) and smaller conidia (5–7.2 × 1.7–2.9 µm) than *P.annesophiae* (4.5–9 × 2–3.5 µm) (Crous et al. 2017), *P.synnematica* (4–11 µm long) (Boonmee et al. 2021) and *P.siamensis* (3–4 µm wide) (Table 2). In addition, it can be differentiated from *P.filiforms* by the indeterminate asexual morph of the latter species (Luo et al. 2019).

## Analysis

**Phylogenetic analysis**

The aligned matrix for the combined analysis, ITS+LSU+SSU had 3105 bp, including ITS = 509 bp, LSU = 1172 bp and SSU = 1424 bp. No topological conflict exists between the tree generated by ML analysis and the Bayesian tree. The Bayesian tree is shown with BS and PP in Fig. 2. All 15 *Phaeoisaria* species in our analyses form a monophyletic group (BS/PP = 59/1.00). Most importantly, the two collections of *Phaeoisarialaianensis* form an independent lineage with strong support (BS/PP = 100/1.00). This lineage groups with *P.dalbergiae* into a highly supported clade (BS/PP = 98/1.00), which is sister to *P.clematidis* (BS/PP = 54/1.00). After searching of NCBIs GenBank nucleotide database based on a megablast, the ITS sequence of *P.laianensis* was found to share 97.46% similarity with *P.dalbergiae* (CPC 39540) and 96.35% similarity with *P.clematidis* (DAOM 226789). In addition, the sequence has nine different loci from that of *P.dalbergiae* and 15 different loci from that of *P.clematidis*.

## Discussion

In our molecular phylogenetic tree, *Phaeoisaria* consists of 15 species and is supported as a monophyletic group (BS/PP = 59/1.00, Fig. 2). The low ML bootstrap might be due to a large number of unavailable sequences for 13 species in *Phaeoisaria*. However, the independent lineage of *P.laianensis* (BS/PP = 100/1.00, Fig. 2) is established and groups with *P.dalbergiae* into a highly supported clade (BS/PP = 98/1.00, Fig. 2). This clade is sister to the four collections of *P.clematidis* although with lower support (BS/PP = 54/1.00, Fig. 2). In addition, the morphological characters of *P.laianensis* can be effortlessly distinguished from *P.dalbergiae* and *P.clematidis* and other species in *Phaeoisaria* (Tables 2, 3). Notably, our results favour *P.laianensis* as a new species in the genus. However, molecular data for *Phaeoisaria* species require enriching to clarify more species relationships in the genus.

*Phaeoisaria* predominantly occurs on leaves, barks, decaying wood and twigs of plants from the freshwater or terrestrial habitats (Table 3), while some are isolated from surface marine sediments (e.g. *P.sedimenticola*, Cheng et al. 2014), some from soil (e.g. *P.annesophieae*, Crous et al. 2017) and some from saprobic decaying fruits (e.g. *P.siameneis*, Hyde et al. 2019). Consequently, the habitats of *Phaeoisaria* are various. In this research, we introduce another lignicolous freshwater fungus, *P.laianensis*, discovered in China and it is noteworthy that the freshwater in which this species exists has been somewhat polluted. *Phaeoisaria* is thought to play an important role in nutrient and carbon cycling, biological diversity and ecosystem functioning of freshwater ecosystems, for their ability to decompose lignocellulose in woody litter, softening the wood and releasing nutrients (Bucher et al. 2004, Vijaykrishna et al. 2005, Hyde et al. 2016, Luo et al. 2018). Nonetheless, some *Phaeoisaria* species are pathogenic to humans, for example, it has been reported that *P.clematidis* and *Phaeoisaria* sp. can cause corneal inflammation of the eye (keratitis) (Guarro et al. 2000, Chew et al. 2010) and the former species is saprotrophic, which is similar to *P.laianensis*. What is the role of *P.laianensis* in ecosystem functioning? Is this species also pathogenic to humans? Such questions are waiting to be investigated by researchers.

## Supplementary Material

XML Treatment for Phaeoisaria laianensis

## Figures and Tables

**Table 1.** Sequences used in this study. **Note**: Ex-type strains are in **bold**. The sequences of new species are indicated as underlined and unavailable sequences in GenBank are indicated by hyphen "-".

Taxonomy	Strain	GenBank accession numbers		
		ITS	LSU	SSU
***Adelosphaeriacatenata***	**CBS 138679**	**KT278721**	**KT278707**	**KT278692**
*Ascotaiwaniafusiformis*	MFLUCC 15-0625	–	KX550894	KX550898
***Ascotaiwaniafusiformis***	**MFLU 15-1156**	**MG388215**	**NG–057114**	–
*Ascotaiwanialignicola*	NIL 00005	HQ446341	HQ446364	HQ446284
*Ascotaiwaniasawadae*	SS00051	HQ446340	HQ446363	HQ446283
***Bactrodesmiastrumobovatum***	**FMR 6482**	**FR870264**	**FR870266**	–
***Bactrodesmiastrumpyriforme***	**FMR 10747**	**FR870263**	**FR870265**	–
*Brachysporiellasetosa*	HKUCC 3713	–	AF132334	–
*Canalisporiumexiguum*	SS 00809	GQ390296	GQ390281	GQ390266
***Canalisporiumgrenadoideum***	**BCC 20507**	–	**GQ390267**	**GQ390252**
*Canalisporiumpulchrum*	SS03982	GQ390292	GQ390277	GQ390262
*Conioscyphalignicola*	CBS 335.93	–	AY484513	JQ437439
***Conioscyphaminutispora***	**CBS 137253**	–	**MH878131**	–
***Conioscyphaperuviana***	**CBS 137657**	–	**KF781539**	–
*Conioscyphavaria*	CBS 113653	–	AY484512	AY484511
***Fuscosporellapyriformis***	**MFLUCC 16-0570**	**MG388217**	**KX550896**	**KX550900**
*Helicoonfarinosum*	DAOM 241947	JQ429145	JQ429230	–
*Leotialubrica*	AFTOLID 1	DQ491484	AY544644	AY544746
*Melanotrigonumovale*	CBS 138815	KT278722	KT278711	KT278698
*Melanotrigonumovale*	CBS 138744	KT278725	KT278710	KT278697
*Melanotrigonumovale*	CBS 138743	KT278724	KT278709	KT278696
***Melanotrigonumovale***	**CBS 138742**	**KT278723**	**KT278708**	**KT278695**
*Microglossumrufum*	OSC100641	–	DQ470981	DQ471033
***Mucisporaobscuriseptata***	**MFLUCC 15-0618**	**MG388218**	**KX550892**	**KX550897**
***Parafuscosporellamoniliformis***	**MFLUCC 15-0626**	**MG388219**	**KX550895**	**KX550899**
***Phaeoisariaannesophieae***	**CBS 143235**	**MG022180**	**MG022159**	–
*Phaeoisariaannesophieae*	MFLU190531	MT559109	MT559084	–
***Phaeoisariaaquatica***	**MFLUCC 16-1298**	**MF399237**	**MF399254**	–
*Phaeoisariaclematidis*	MFLUCC 16-1273	MF399229	MF399246	–
*Phaeoisariaclematidis*	MFLUCC 17-1341	MF399230	MF399247	MF399216
*Phaeoisariaclematidis*	MFLUCC 17-1968	MG837022	MG837017	MG837027
*Phaeoisariaclematidis*	DAOM 226789	JQ429155	JQ429231	JQ429243
***Phaeoisariadalbergiae***	**CPC 39540**	**OK664703**	–	–
***Phaeoisariafasciculata***	**CBS 127885**	**KT278719**	**KT278705**	**KT278693**
*Phaeoisariafasciculata*	DAOM 230055	KT278720	KT278706	KT278694
***Phaeoisariafiliformis***	**MFLUCC 18-0214**	**MK878381**	**MK835852**	**MK834785**
*Phaeoisariaguttulata*	MFLUCC 17-1965	MG837021	MG837016	MG837026
***Phaeoisarialaianensis***	**CCTCC AF 2022069**	**ON937559**	**ON937557**	**ON937562**
*Phaeoisarialaianensis*	CCTCC AF 2022073	ON937560	ON937561	ON937558
***Phaeoisarialoranthacearum***	**CBS 140009**	**KR611888**	**MH878676**	–
*Phaeoisarialoranthacearum*	BYCDW25	MG820097	–	–
*Phaeoisarialoranthacearum*	BYCDW24	MG820098	–	–
*Phaeoisariamicrospora*	MFLUCC 16-0033	MF671987	MF167351	–
***Phaeoisariapseudoclematidis***	**MFLUCC 11-0393**	**KP744457**	**KP744501**	**KP753962**
***Phaeoisariasedimenticola***	**CGMCC3.14949**	**JQ074237**	**JQ031561**	–
*Phaeoisariasedimenticola*	S-908	MK878380	MK835851	–
***Phaeoisariasiamensis***	**MFLUCC 16-0607**	**MK607610**	**MK607613**	**MK607612**
*Phaeoisariasparsa*	FMR 11939	–	HF677185	–
***Phaeoisariasynnematica***	**NFCCI 4479**	**MK391494**	**MK391492**	–
*Phragmocephalastemphylioides*	KAS 4277	KT278730	KT278717	–
***Plagiascomafrondosum***	**CBS 139031**	–	**KT278713**	**KT278701**
***Pleurotheciellacentenaria***	**DAOM 229631**	**JQ429151**	**JQ429234**	**JQ429246**
*Pleurotheciellarivularia*	CBS 125237	JQ429161	JQ429233	JQ429245
***Pleurotheciellarivularia***	**CBS 125238**	**JQ429160**	**JQ429232**	**JQ429244**
*Pleurotheciellauniseptata*	KUMCC 15-0407	MF399231	MF399248	–
***Pleurotheciumaquaticum***	**MFLUCC 17-1331**	**MF399245**	**MF399263**	–
*Pleurotheciumaquaticum*	MFLUCC 21-0148	OM654775	OM654772	OM654807
*Pleurotheciumfloriforme*	MFLUCC 15-0628	KY697281	KY697277	KY697279
*Pleurotheciumobovoideum*	CBS 209.95	EU041784	EU041841	–
*Pleurotheciumpulneyense*	MFLUCC 16-1293	–	MF399262	MF399228
*Pleurotheciumrecurvatum*	CBS 138747	KT278728	KT278714	KT278703
*Pleurotheciumrecurvatum*	CBS 131272	JQ429149	JQ429237	JQ429251
*Pleurotheciumrecurvatum*	CBS 101581	JQ429148	AF261070	JQ429248
*Pleurotheciumsemifecundum*	CBS 131482	JQ429158	JQ429239	JQ429253
***Pleurotheciumsemifecundum***	**CBS 131271**	**JQ429159**	**JQ429240**	**JQ429254**
*Savoryellalongispora*	SAT 00322	HQ446359	HQ446380	HQ446302
*Savoryellapaucispora*	SAT 00866	–	HQ446381	HQ446303
*Savoryellaverrucosa*	SS 00052	HQ446353	HQ446374	HQ446296
*Sterigmatobotrysmacrocarpa*	DAOM 230059	JQ429154	GU017316	–
*Sterigmatobotrysmacrocarpa*	PRM 915682	JQ429153	GU017317	JQ429255
*Sterigmatobotrysrudis*	DAOM 229838	JQ429152	JQ429241	JQ429256

**Table 2.** Synopsis 1 of asexual morphological characteristics of *Phaeoisaria* species. **Note**: Hyphens “-” are indeterminate or unavailable data.

Species	Synnemata (µm)	Synnemata characteristics	Conidiophores (µm)	Conidiophores characteristics	Conidia (µm)	References
*Phaeoisarialaianensis*	290‒848 × 9.3‒30.7	Erect, rigid, dark brown to black, velvety, smooth, composed of compactly and parallel adpressed conidiophores	116.2‒491.1 × 2‒3.2	Macronematous, synnematous, septate, branched, brown to dark brown, smooth	5‒7.2 × 1.7‒2.9	This study
*P.aguilerae*	-	-	-	-	18–29.5 × 4–5	Castañeda Ruiz et al. (2002)
*P.annesophieae*	-	-	Conidiophores indeterminate	Sometimes grouping in strands of 2–4 hyphae, a rising from aerial hyphae, cylindrical, hyaline to pale brown	4.5–9 × 2–3.5	Crous et al. (2017)
*P.aquatica*	-	Erect, rigid, dark brown to black, velvety, smooth	1028–1262 × 3.5–4.5	Macronematous, synnematous, brown to dark brown, smooth	6.5–7.5 × 2.5–3.5	Luo et al. (2018)
*P.bambusae*	-	Erect, rigid, dark brown toblack, velvety, smooth	-	Macronematous, synnematous, brown to dark brown, smooth	-	Höhnel (1909), Hyde et al. (2019), Luo et al. (2019), Réblová et al. (2016)
*P.caffra*	-	Synnemata composed of at least 10 adpressed hyphae	-	Conidiophores not tuberculate	7.5–12 × 2.5–3.5	Castañeda Ruiz et al. (2002), de Hoog and Papendorf (1976)
*P.clavulata*	-	Stiff synnemata, composed of parallel hyphae, packed with slender, curved conidiogenous cells with very thin, fragile conidiogenous rachides	-	-	1–2 long	Castañeda Ruiz et al. (2002), de Hoog and Papendorf (1976), Mason and Ellis (1953)
*P.clematidis*	1000–1500 × 20–80	Conidiomata scattered, indeterminate, erect, rigid, superficial, dark brown composed of compact appressed conidiophores	312–568 × 2.5–3.5	Macronematous, septate, branched, brown to dark brown, smooth	4–10 × 1.5–2.5	Castañeda Ruiz et al. (2002), Hughes (1958), Luo et al. (2018)
*P.curvata*	-	-	Conidiophores indeterminate	-	(4–)6–8(–11) × (1–)2–3	de Hoog and Papendorf (1976)
*P.dalbergiae*	-	-	10–50 × 1.5–2.5	Indeterminate, erect, subcylindrical, hyaline, smooth, 0–2-septate, unbranched or branched at apex	0.5 µm diam, (5 –)6–7 × (1.5–)2	Crous et al. (2021)
*P.fasciculata*	-	Synnemata absent	25–65 × 3.0–3.5	Macronematous, arising from brown, thick-walled cells, cylindrical, pale brown, subhyaline towards the apex, unbranched, smooth-walled	6.0–8.0 (–9.0) × 2.0	Réblová et al. (2016)
*P.filiformis*	-	-	-	-	-	Luo et al. (2019)
*P.glauca*	-	-	Conidiophores indeterminate	-	2.5–3.5 × 1.6–2.2	de Hoog and Papendorf (1976)
*P.guttulata*	-	Erect, rigid, dark brown to black, velvety, smooth, composed of compactly and parallel adpressed conidiophores	480–700 × 2–5	Macronematous, synnematous, erect, septate, smooth, mid-brown to dark brown	3.5–5.5 × 2.5–4.8	Hyde et al. (2018)
*P.infrafertilis*	-	Synnemata narrow, composed of only 5-6 brown adpressed hyphae	-	-	19.5–22 × 2–3	de Hoog and Papendorf (1976), Sutton and Hodges (1976)
*P.loranthacearum*	-	-	10–30 × 2–3	Arising from superficial hyphae, erect, solitary, branched at base or not, subcylindrical, straight to geniculate-sinuous, 1–3-septate, hyaline	(5)7– 8(9) × (1.5) 2(3)	Crous et al. (2015)
*P.magnifica*	-	Synnemata brush-like, synnemata with flaring hyphae at the tip	-	Growing well away from the column in the apical portion	5–6.5 × 4–4.5	de Hoog and Papendorf (1976), Deighton (1974)
*P.microspora*	35–238 µm long, 4–31 µm wide at the base, 5–35 µm wide at the apex	Erect, straight or flexuous, dark brown at base, pale brown at apex	25–225 × 1–3	Macronematous, synnematous, septate, branched at the apex, smooth, pale to dark brown	4.5–6.9 × 1.3–3.1	Hyde et al. (2017)
*P.muscariformis*	-	-	-	-	12–22 × 4	Castañeda Ruiz et al. (2002), Siboe et al. (1999)
*P.pseudoclematidis*	200–500 µm long, 40–80 µm wide at the base, 40– 60 µm wide in the middle, 20–30 µm wide at the apex	Erect, rigid, dark brown, velvety, smooth, composed of compactly and parallel adpressed conidiophores	50–500 × 2–3	Macronematous, synnematous, brown to dark brown, septate, branched, smooth	5–8.5 × 3–4	Liu et al. (2015)
*P.sedimenticola*	up to 4000 µm high or sometimes longer, 70– 90 µm wide	Erect, cylindrical to subulate, consisting of very regular, parallel, brown hyphae			aseptate (3.5–)4.5–5.5(–7.5) × (2.5–)3–4(–4.5) 1-septate (4.5–)5.5–6.5(–9) × (2–)2.5–3.5(–4.5)	Cheng et al. (2014)
*P.siamensis*	330–380 × 20–25(–30)	Conidiomata scattered, indeterminate, erect, rigid, superficial, dark brown composed of compactly appressed conidiophores	2–2.5(–3) µm wide	Macronematous, in synnematous conidiomata, scattered, synnemata subulate or cylindrical, indeterminate, at the base 13–15 µm beneath the fertile portion with conidiogenous cells, composed of medium to dark brown, smooth, septate parallel hyphae, splaying out at the middle to apex	5–8 × 3–4	Hyde et al. (2019)
*P.sparsa*	-	Synnemata composed of at least 10 adpressed hyphae	-	Not tuberculate	10–15.5 × 2.5–3.5	de Hoog and Papendorf (1976), Sutton (1973)
P.sparsavar.cubensis	-	-	-	-	(4–)7– 11(–17) ×(1.5–) 2– 3(–4)	Mercado-Sierra et al. (1997), Mel’nik (2012)
*P.synnematica*	399–960 × 12–30	Synnematal, erect, rigid, dark brown to olivaceous brown, composed of compactly parallel appressed conidiophores, cylindrical to clavate	1.5–960 × 1–3.5	Macronematous to semi- macronematous, highly geniculate, dark brown to olivaceous brown, synnematous, simple to dichotomously branched, emerging out at the apex and along the sides of the upper half or two thirds of each synnema, dark brown at the base, brown to pale brown	4–11 × 2–5	Boonmee et al. (2021)
*P.tuberculata*	-	Synnemata composed of at least 10 adpressed hyphae	-	Conspicuously tuberculate	8–13.5 × 1.5–2	Castañeda Ruiz et al. (2002), Sutton (1993)
*P.uniseptata*	-	-	-	-	(3.5–) 5.5– 7.5 (–10) × 1.5–3	de Hoog and Papendorf (1976), Mercado-Sierra (1984), Mel’nik (2012)
*P.vietnamensis*	330–380 µm high, 20–25(– 30) µm wide at the base	-	2–2.5(–3) µm wide	Macronematous, in synnematous conidiomata, scattered, synnemata subulate or cylindrical, indeterminate composed of medium to dark brown, smooth, septate parallel hyphae	18.5– 23.5 × 4.5–5	Mel’nik (2012)

**Table 3.** Synopsis 2 of asexual morphological characteristics of *Phaeoisaria* species. **Note**: Hyphens "-" are indeterminate or unavailable data.

Species	Conidia septation	Conidia characteristics	Host	District	References
*Phaeoisarialaianensis*	Aseptate	Ellipsoidal to obovoid, straight, rounded at the apex, obtuse and tapering towards base, hyaline, guttulate, smooth-walled	Decaying wood	China, Anhui Province	This study
*P.aguilerae*	1-septate, rarely 2–3-septate	Clavate or cylindrical, curved, with obtuse, rounded apex, slightly uncinate, and truncate base, hyaline, smooth	Decaying twig submerged in river	Cuba	Castañeda Ruiz et al. (2002)
*P.annesophieae*	Aseptate	Ellipsoidal to obovoid, straight or slightly curved, rounded at the ends or sometimes tapering towards the base, hyaline, guttulate, smooth-walled	Isolated from soil	The Netherlands, Geldermalsen	Crous et al. (2017)
*P.aquatica*	Aseptate	Ellipsoidal to obovoidal, rounded at the apex, hyaline, with two guttules smooth-walled	Decaying wood submerged in Jinsha River	China, Yunnan Province	Luo et al. (2018)
*P.bambusae*	aseptate or septate	Ellipsoidal to obovoidal, fusiform-cylindrical to falcate, hyaline, straight, guttulate, smooth-walled	Unidentified submerged bamboo	Indonesia	Höhnel (1909), Hyde et al. (2019), Luo et al. (2019), Réblová et al. (2016)
*P.caffra*	Aseptate, rarely 1-sepate	Conidia straight, ellipsoidal to clavate, obovoid, not attenuated at the apex, pale yellow brown, smooth	On decaying leaf of *Podocarpus*	Cape Province	Castañeda Ruiz et al. (2002), de Hoog and Papendorf (1976)
*P.clavulata*	Aseptate	Broadly ellipsoidal to ± spherical, subspherical, smooth, hyaline	On rotten decorticated wood	Great Britain	Castañeda Ruiz et al. (2002), de Hoog and Papendorf (1976), Mason and Ellis (1953)
*P.clematidis*	Aseptate	Obovoidal, rounded at the apex, obtuse and tapering towards base, hyaline, smooth-walled	Decaying wood submerged in Lancang River	China, Yunnan Province	Castañeda Ruiz et al. (2002), Hughes (1958), Luo et al. (2018)
*P.curvata*	Aseptate	Smooth, thin-walled, hyaline, clavate to obovoid and pointed at base, curved, occasionally sickle-shaped	Rotten leaves of *Parinaricapensis*	South West Africa	de Hoog and Papendorf (1976)
*P.dalbergiae*	Aseptate	Solitary, hyaline, smooth, thin-walled, guttulate, subcylindrical to obovoid, tapering towards both ends, apex subobtuse, base with truncate hilum	On bark of *Dalbergiaarmata*	South africa, Northern Province	Crous et al. (2021)
*P.fasciculata*	Aseptate	Ellipsoidal to obovoid, straight, rounded at the apex, obtuse and tapering towards base, hyaline, smooth-walled	Decorticated wood of *Sambucusnigra*	Canada, Ontario, Goulbourn Twp	Réblová et al. (2016)
*P.filiformis*	-	-	Decaying wood submerged in freshwater stream	Thailand, Sai khu Waterfall	Luo et al. (2019)
*P.glauca*	Aseptate	Smooth, thin-walled, hyaline, guttuliform to ellipsoidal,with pointed base, occasionally sickle-shaped	On rotten wood of *Quercus* sp.	America, Newfield	de Hoog and Papendorf (1976)
*P.guttulata*	Aseptate	Globose to obovoid, hyaline, smooth-walled, guttulate	Decaying wood submerged in Suoluo River	China, Guizhou Province	Hyde et al. (2018)
*P.infrafertilis*	Aseptate, rarely 1-sepate	Conidia falcate,hyaline	On dead leaves of *Eucalyptus*	Brazil	de Hoog and Papendorf (1976), Sutton and Hodges (1976)
*P.loranthacearum*	-	Solitary, hyaline, smooth, fusoidal-ellipsoidal with obtuse ends, straight to falcate, guttulate	On twigs of *Loranthuseuropaeus*	Germany	Crous et al. (2015)
*P.magnifica*	Aseptate	Straight, ellipsoidal to obovo1d, clavate, very pale olivaceous, smooth	On *Bambusa*	New Caledonia	de Hoog and Papendorf (1976), Deighton (1974)
*P.microspora*	Aseptate	Solitary, fusiform, straight, smooth-walled, guttulate, hyaline	On decaying wood	Thailand, Krabi, Wat ThumSua	Hyde et al. (2017)
*P.muscariformis*	3-sepate	Cylindrical-fusiform, subhyaline, smooth	On leaves of *Tiliacorakenyensis*	Kenya	Castañeda Ruiz et al. (2002), Siboe et al. (1999)
*P.pseudoclematidis*	Aseptate	Cylindrical-ovate, straight, hyaline, smooth-walled, guttulate	On dead culm of bamboo (Bambusae)	Thailand, Chiang Rai	Liu et al. (2015)
*P.sedimenticola*	Aseptate, 1-septate	Smooth-walled, hyaline, with a pointed base, usually aseptate when attached to the conidiogenous cells, 0–1-septate after release; aseptate conidia, obovoid to ellipsoidal; 1-septate conidia, obovoid, slightly constricted at septum	Isolated from surface of marine sediment in intertidal zone	China, Shandong Province	Cheng et al. (2014)
*P.siamensis*	Aseptate	Globose to subglobose, hyaline	Saprobic on decaying fruits	Thailand, Chiang Mai Province	Hyde et al. (2019)
*P.sparsa*	0-3-septate	Fusiform to clavate, conidia straight, ellipsoidal to fusiform, hyaline, not attenuated at the apex	On bark of *Acerspicatum*	Saskatchewan	de Hoog and Papendorf (1976), Sutton (1973)
P.sparsavar.cubensis	0–1(–4)-septate	Fusiform, cylindrical or clavate, hyaline, sometimes slightly curved	On dead branch	Cuba	Mercado-Sierra et al. (1997), Mel’nik (2012)
*P.synnematica*	0–1-septate	Dimorphic, clavate to ellipsoidal, cylindrical to falcate, base narrowly truncate, tip obtuse, variable in size, sometimes constricted near septa, 1–2-guttulate, hyaline, smooth-walled	Dead bark of *Azadirachtaindica* (Meliaceae)	India, Maharashtra	Boonmee et al. (2021)
*P.tuberculata*	Asepate, rarely 1-sepate	Conidia fusiform, straight, the apex attenuated, hyaline, smooth, guttulate	On Labiatae	Malawi	Castañeda Ruiz et al. (2002), de Hoog and Papendorf (1976), Sutton (1993)
*P.uniseptata*	Mostly with a median septum	Two-celled, fusiform, ellipsoid, hyaline, cylindrical or clavate	On dead branch	Cuba	de Hoog and Papendorf (1976), Mercado-Sierra (1984), Mel’nik (2012)
*P.vietnamensis*	A single median septum	Fusiform-subcylindrical to short obovoid-subclavate, somewhat attenuated towards the base, apex obtuse, straight to slightly curved, not constricted, hyaline, smooth, often guttulate	On bark of a living unidentified liane	South Vietnam, Dong Nai Province	Mel’nik (2012)
